# Risk of Microangiopathy in Patients with Epilepsy under Long-term Antiepileptic Drug Therapy

**DOI:** 10.3389/fneur.2018.00113

**Published:** 2018-03-12

**Authors:** Nai-Ching Chen, Chih-Hsin Chen, Tsu-Kung Lin, Shang-Der Chen, Meng-Han Tsai, Chiung-Chih Chang, Wan-Chen Tsai, Yao-Chung Chuang

**Affiliations:** ^1^Department of Neurology, Kaohsiung Chang Gung Memorial Hospital, Chang Gung University College of Medicine, Kaohsiung, Taiwan; ^2^Department of Ophthalmology, Kaohsiung Chang Gung Memorial Hospital, Chang Gung University College of Medicine, Kaohsiung, Taiwan; ^3^Institute for Translational Research in Biomedicine, Kaohsiung Chang Gung Memorial Hospital, Chang Gung University College of Medicine, Kaohsiung, Taiwan; ^4^Department of Neurology, Faculty of Medicine, College of Medicine, Kaohsiung Medical University, Kaohsiung, Taiwan; ^5^Department of Biological Science, National Sun Yat-sen University, Kaohsiung, Taiwan

**Keywords:** antiepileptic drug therapy, atherosclerosis, microangiopathy, retinopathy, microalbuminuria

## Abstract

**Background:**

Long-term antiepileptic drug (AED) therapy is considered a risk factor of atherosclerosis. Furthermore, the duration of therapy contributes to acceleration of large-vessel atherosclerosis. Therefore, in this study, we tested the hypothesis that long-term AED therapy plays a crucial role in the pathogenesis of microangiopathy in patients with epilepsy.

**Methods:**

We recruited 120 patients with epilepsy (age, 18–60 years) and 40 healthy controls. Optical coherence tomography (OCT) was used to measure the central macular thickness and diameters of the retinal artery and vein to evaluate atherosclerotic retinopathy; microalbumin and creatinine levels in urine were assessed to evaluate atherosclerotic nephropathy. In addition, high-sensitivity C-reactive protein (hs-CRP), lipid profiles, homocysteine, folate, uric acid, and body mass index were determined.

**Results:**

The ratio of urine albumin to creatine and OCT findings showed that patients with epilepsy had higher abnormal microalbuminuria and narrowing retinal vein diameters, respectively. Multiple linear regression analysis showed that increased triglyceride and hs-CRP levels might contribute to microalbuminuria. In addition, serum creatinine, duration of AED therapy, enzyme-inducing AED therapy, and duration of enzyme-inducing AED therapy were candidate risk factors for retinal vein narrowing.

**Conclusion:**

Patients with epilepsy are at a higher risk for microangiopathy presented as retinopathy and nephropathy. Long-term AED therapy, particularly with enzyme-inducing AEDs; high triglyceride levels, and inflammatory processes play an important role in the development of microangiopathy in patients with epilepsy.

## Introduction

More than 30% of patients with epilepsy do not experience seizure remission despite appropriate and adequate antiepileptic drug (AED) therapy (1). Therefore, long-term or lifelong AED treatment is often required for those patients with intractable epilepsy. Prolonged AED therapy, particularly with enzyme-inducing AEDs (EIAEDs), may promote the pathogenesis of atherosclerosis and increase atherosclerotic risk arising from cerebrovascular events or ischemic heart disease resulting in high morbidity and mortality in patients with epilepsy (2–5).

Atherosclerosis is a chronic systemic condition involving the wall of the arteries and multiple factors account for the pathogenesis and progress of atherosclerosis (6). We have previously shown (3, 5) that the duration of AED therapy is an important independent factor affecting the acceleration of atherosclerosis by increasing the common carotid artery intima media thickness (CCAIMT) and metabolic disturbances. These results suggest that the cumulative effects of long-term treatment with AEDs play a crucial role in the pathogenesis of atherosclerosis-related macroangiopathy in large blood vessels. Long-term treatment with AEDs may also cause microangiopathy in target organs, such as the retina and kidneys, which may lead to atherosclerosis-related retinopathy and nephropathy. Microangiopathy has been found to be associated with diabetes mellitus, hypertension, and other conditions that increase the risk of cerebrovascular diseases (7–10); however, microvascular complications present in patients with epilepsy under prolonged AED therapy remains unclear.

Microangiopathies, including retinal proliferation and microalbuminuria, have been reported as indicators of atherosclerotic retinopathy and nephropathy (8–11). However, the reported incidence of retinopathy and nephropathy in patients with epilepsy is limited. To investigate atherosclerosis-related microangiopathy in retinal vessels, optical coherence tomography (OCT) has been used for assessing atherosclerotic plaques in the walls of the retinal blood vessels (12, 13). In addition, biomarkers of kidney function, including serum creatinine, estimated glomerular filtration rate (eGFR), and microalbuminuria, can predict atherosclerosis-related nephropathy (8, 9, 14). Recent studies have shown that microalbuminuria may represent endothelial dysfunction in renal vessels and may be associated with increased risk of atherosclerosis-related renal microangiopathy (8–10, 14).

This study evaluates the hypothesis that prolonged treatment with AEDs may play a crucial role in the pathogenesis of microvascular endothelial cell damage in the end organs, particularly in the kidney and retina, which result in atherosclerosis-related retinopathy and nephropathy in patients with epilepsy.

## Materials and Methods

### Study Design

This is a case-control study conducted at a single center. The study hospital, Kaohsiung Chang Gung Memorial Hospital, is a tertiary medical center in southern Taiwan. Ethical approval for this study (Ethical Committee NO100-2011B) was provided by the Chang Gung Medical Foundation Institutional Review Board, and written informed consent was obtained from both the patients as well as from healthy controls. All procedures performed in this study involving human participants were in accordance with the 1964 Helsinki declaration and its later amendments or comparable ethical standards.

### Subjects

From October 2011 to September 2013, 120 patients (aged between 18 and 65 years) who received AED treatment for more than 2 years were enrolled through the Epilepsy Outpatient Clinic of Kaohsiung Chang Gung Memorial Hospital. Forty healthy volunteers who received an annual physical checkup were recruited as normal controls. Patients who had discontinued their medication within 2 years were excluded from this study. The other exclusion criteria for patients and controls included cerebrovascular diseases; ischemic heart diseases; hypertension; diabetes mellitus; clinical history of retinopathy; hematologic, endocrine, and autoimmune diseases; and nephritis, nephrotic syndrome, and chronic kidney disease (3, 5). Patients with a documented diagnosis of gouty arthritis, manifestation of tophi, or nephrolithiasis associated with hyperuricemia were also excluded. We also excluded patients who consumed vitamins, tobacco, antihypertensive drugs, oral hypoglycemic agents, or those who received medication that affected the metabolism of uric acid or lipids. Meanwhile, patients with retinal vascular abnormalities, such as diabetic retinopathy, hypertensive retinopathy, retinal vein occlusion, age-related macular degeneration, and other retinal vascular disorders, were excluded.

All patients were subjected to a general physical and neurological examination as well as clinical interviews. Clinical demographic data, including the age at onset of seizures, seizure types, etiology of epilepsy, seizure frequency (per month), duration of AED therapy, and any current AEDs therapy were recorded. To identify any confounding high-risk factors for atherosclerosis and to exclude epilepsy that arises from asymptomatic cerebrovascular diseases, magnetic resonance imaging of the brain was conducted for all the patients.

The AEDs being used by the enrolled patients included lamotrigine, carbamazepine, phenytoin, valproate acid, topiramate, phenobarbital, levetiracetam, vigabatrin, and gabapentin. Carbamazepine, phenytoin, and phenobarbital have been defined as enzyme inducers of the liver cytochrome P450 (CYP450) system (3, 11). Lamotrigine, levetiracetam, valproate, topiramate, vigabatrin, and gabapentin are considered non-enzyme inducers (3, 11). We defined the duration of EIAED therapy as the time period during which patients received continuous treatment with enzyme inducers. The duration of non-EIAED therapy was defined as the time period during which patients received sustained treatment with or switched to non-enzyme inducers.

### Blood Collection and Analysis of Circulating Biochemical Markers

After overnight fasting, blood samples were obtained from the patients between 8 and 10 a.m. and analyzed at the Central Laboratory of Kaohsiung Chang Gung Memorial Hospital for serum levels of total cholesterol, high-density lipoprotein cholesterol (HDL-C), low-density lipoprotein cholesterol (LDL-C), triglycerides, folate, homocysteine, blood glucose, glycated hemoglobin (HbA1c), high-sensitive C-reactive protein (hs-CRP), uric acid, and creatinine levels. All circulating biochemical markers were analyzed according to our previous reports (3, 5, 15).

Estimated glomerular filtration rate was calculated as:

Male: [(140−age)×body weight]/(serum creatinine×72)Female: [(140−age)×body weight]/(serum creatinine×72)×0.85.

### Measurement of Microalbuminuria

Urine samples obtained from the patients were analyzed for creatinine and microalbuminuria levels. The ratio of albumin to creatinine levels was also calculated. Microalbuminuria was defined as a ratio of urine albumin to urine creatinine of 30–300 g/mg (16). Routine urinary analysis was also conducted to exclude urinary tract infections and other etiologies of abnormal microalbuminuria.

### Assessment of Retinal Microangiopathy

Retinal microangiopathy was measured by a third-generation OCT (Model 3000, Carl-Zeiss Instruments, Dublin, CA, USA). OCT using a manually assisted technique was conducted for all the patients by using a previously reported methodology (17, 18) by experienced examiners in order to measure the central macular thickness, retinal artery diameter, and retinal vein diameter (17, 18). We routinely measured the diameter of the retinal artery and vein over the superior-temporal arcade one-disk distance away from the upper disk margin.

### Statistical Analysis

Three separate statistical analyses were performed. First, gender and microalbumiuria (defined as urine albumin/creatinine ratio ≥30) were compared between patients with epilepsy and controls by using a Chi-square test. Comparisons of continuous variables with normal distribution between the two study groups were made using the Student’s *t-*test. Comparison of continuous variables without normal distribution between the two study groups were made using the Mann–Whitney Test. Second, correlation analysis was used to evaluate the relationship between urine albumin/creatinine ratio; vein diameter; and variables that included age, body mass index (BMI), duration of AED therapy, duration of EIAED and non-EIAED, number and type of AED therapy, lipid profile, hs-CRP, folate, homocysteine, HbA1c, and serum creatinine levels. Third, factors with *p* value <0.3 from the correlation analysis were used to evaluate the relationships between baseline clinical factors associated with the urine albumin/creatinine ratio and vein diameter by using the stepwise method of multiple linear regression. All statistical analysis was conducted using the Statistical Package for Social Sciences software package (version 13 for Windows^®^, SPSS Inc., Chicago, IL, USA).

## Results

### Characteristics and Demographic Data of Patients with Epilepsy

The characteristics and demographic data of 120 patients with epilepsy are listed in Table 1. The age at onset of epilepsy ranged from 3 months to 49 years, and the duration of epilepsy ranged from 2 to 57 years. Their average seizure frequency ranged from 0 to 80 per month. The duration of AED therapy at the time of study ranged from 2 to 56 years, and it was significantly correlated to the duration of epilepsy (*p* < 0.001). 57 patients (47.5%) received monotherapy and 63 (52.5%) were on multiple AED therapy. Seventy-three patients with epilepsy received EIAEDs for seizure control. The duration of EIAED therapy in 73 patients at the time of study ranged from 2 to 57 years; the duration of non-EIAED therapy in the other 47 patients ranged from 3 to 28 years. Based on statistical analysis, the duration of therapy did not show significance between the EIAED and non-EIAED groups (*p* = 0.069). According to etiology, 75 patients (62.5%) had idiopathic or cryptogenic epilepsy and 45 patients (37.5%) had symptomatic epilepsy. In terms of basic demographic data, age and gender between patients and controls did not show a significant difference (Table 2).

**Table 1:** Baseline characteristics of 120 patients with epilepsy enrolled in this study.

Characteristics	
Age at onset (years)	21.8 ± 11.7
Duration of epilepsy (years)	18.6 ± 8.1
Type of seizures, *n* (%)	
Generalized	12 (10%)
Partial	108 (90%)
Seizure free,* *n* (%)	51 (42.5%)
Etiology, *n* (%)	
Idiopathic/cryptogenic	75 (62.5%)
Symptomatic	45 (37.5%)
Duration of AED therapy (years)	17.8 ± 10.8
Duration of EIAED therapy (years)	14.5 ± 12.4
Duration of non-EIAED therapy (years)	13.7 ± 6.4
Mode of AED therapy, *n* (%)	
Single	57 (47.5%)
Multiple	63 (52.5%)
EIAEDs	73 (60.8%)

*Values are expressed in mean ± SD*.

*Seizure free for more than 12 months under current AED therapy.

*AED, antiepileptic drug, EIAEDs, enzyme-inducing antiepileptic drugs*.

**Table 2:** Demographic data and vascular risk factors in patients with epilepsy vs. controls.

	Controls (*n* = 40)	Patients (*n* = 120)	*p* Value
Age (years)	38.7 ± 10.6	38.6 ± 9.6	0.936
Gender (female/male)	20/20	60/60	1
Body mass index (kg/m^2^)	23.3 ± 3.4	25.0 ± 4.8*	0.027
Fasting blood sugar (mg/dL)	92.2 ± 7.3	94.9 ± 18.8	0.679
Glycated hemoglobin (HbA1c) (%)	5.5 ± 0.3	5.5 ± 0.7	0.336
Uric acid (mg/dL)	5.6 ± 1.5	5.0 ± 1.7	0.051
Cholesterol (mg/dL)			
Total	169.8 ± 21.8	192.2 ± 32.5*	<0.001
HDL-C	64.3 ± 15.3	67.1 ± 17.9	0.416
LDL-C	89.9 ± 19.9	103.7 ± 27.1*	0.001
Triglyceride (mg/dL)	77.4 ± 39.8	115.1 ± 83.1*	0.003
Homocysteine (umol/L)	12.8 ± 8.5	14.9 ± 10.4*	0.049
Folate (ng/mL)	8.2 ± 2.9	7.1 ± 3.5*	0.012
hs-CRP (mg/L)	1.5 ± 3.2	4.2 ± 9.5*	0.009
Creatinine (mg/dL)	0.8 ± 0.2	0.8 ± 0.2	0.273
Urine albumin/creatinine ratio (mg/g)	7.0 ± 4.5	20.2 ± 50.9*	0.006
Urine albumin/creatinine ratio ≥30, n (%)	0 (0%)	14 (11.7%)*	0.023
Central macular thickness, right eye (μm)	197.7 ± 8.2	197.4 ± 13.0	0.995
Central macular thickness, left eye (μm)	198.4 ± 7.8	198.3 ± 12.7	0.934
Retinal artery diameter, right eye (μm)	65.5 ± 1.6	65.9 ± 2.4	0.458
Retinal artery diameter, left eye (μm)	66.6 ± 2.3	66.5 ± 2.9	0.596
Retinal vein diameter, right eye (μm)	88.3 ± 2.7	86.2 ± 3.6*	0.001
Retinal vein diameter, left eye (μm)	88.6 ± 2.7	86.6 ± 3.8*	0.004

*Values are expressed in mean ± SD*.

*AED, antiepileptic drug, HDL-C, high-density lipoprotein cholesterol, LDL-C, low-density lipoprotein cholesterol, hs-CRP, high-sensitivity C-reactive protein; BMI, body mass index*.

*p < 0.05 vs. normal control.

### Microangiopathy and Vascular Risk Factors Are Increased in Patients with Epilepsy

The retinal artery diameter and central macular thickness on either side of the eyes did not show a significant difference between the patients and controls. Univariate analysis (Table 2) revealed that the retinal vein diameters on either side were significantly narrower in patients with epilepsy. In addition, the urine albumin/creatinine ratio was significantly increased in patients with epilepsy. A urine albumin/creatinine ratio over 30 was not found in the control group; however, 11.7% of patients with epilepsy had a urine albumin/creatinine ratio over 30.

Table 2 also shows that BMI, total cholesterol, LDL-C, triglyceride, homocysteine, and hs-CRP were significantly increased, and folate was decreased in patients with epilepsy. Other results included fasting blood sugar, uric acid, HDL-C, and serum creatinine did not show significant differences between the patient and control groups.

### Risk Factors Associated with Microangiopathy

The patients with epilepsy were further divided into two groups based on whether or not they received long-term EIAED therapy. Based on the statistical analysis, uric acid (*p* = 0.007), total cholesterol (*p* = 0.004), HDL-C (*p* = 0.003), and urine albumin/creatinine ratio (*p* = 0.009) showed significant differences between the two patient subgroups. Other factors, such as age, gender, fasting blood sugar, glycated hemoglobin, and bilateral retinal artery and vein diameter did not show significant differences between the patient subgroups.

Correlation analysis (Table 3) revealed that the urine albumin/creatinine ratio was significantly correlated with blood levels of EIAEDs, hs-CRP, total cholesterol, LDL-C, and triglycerides. Moreover, bilateral retinal vein diameter showed a significant correlation with age, serum creatinine, EIAED therapy, duration of AED therapy, and duration of EIAED therapy.

**Table 3:** Correlation analysis of the effects of candidate risk factors on microangiopathy in patients with epilepsy and controls.

	Urine albumin/creatinine ratio		Retinal vein diameter	
Variables	Correlation coefficient	*p* Value	Correlation coefficient	*p* Value
Age	−0.080	0.195	−0.221*	0.008
Body mass index	0.058	0.266	0.055	0.277
Duration of AED therapy	0.030	0.374	−0.250*	0.003
EIAEDs	−0.162*	0.040	0.155*	0.047
Duration of EIAED therapy	0.080	0.193	−0.327*	0.001
Duration of non-EIAED therapy	−0.085	0.179	−0.034	0.359
AED number	0.040	0.333	−0.020	0.416
Homocysteine	0.004	0.482	0.109	0.119
hs-CRP	0.206*	0.013	0.037	0.345
Total cholesterol	0.217*	0.009	−0.027	0.386
LDL-C	0.167*	0.036	0.045	0.315
Triglyceride	0.313*	0.001	0.083	0.187
Glycated hemoglobin (HbA1c)	0.084	0.182	−0.021	0.410
Creatinine	−0.044	0.318	0.272*	0.001

*AED, antiepileptic drug, EIAEDs, enzyme-inducing antiepileptic drugs, hs-CRP, high-sensitivity C-reactive protein; LDL-C, low-density lipoprotein cholesterol; BMI, body mass index*.

*The duration of AED therapy was considered 0 in the control group*.

*p < 0.05.

Variables with *p* < 0.03 were further analyzed by a multiple linear regression test to identify the factors that promote retinal and renal microangiopathy in patients with epilepsy. Multiple linear regression analysis showed that hs-CRP and triglyceride levels were significantly correlated to the urine albumin/creatinine ratio (Table 4). In addition, creatinine, EIAED therapy, duration of AED therapy, and duration of EIAED therapy were significantly correlated with retinal vein diameter.

**Table 4:** Multiple linear regression analysis of association of microangiopathy with duration of AED therapy and other candidate risk factors.

	Urine albumin/creatinine ratio			Retinal vein diameter		
Variables	*B*	SE	*p* Value	*B*	SE	*p* Value
Age	−0.119	−1.366	0.175	−0.117	−1.373	0.173
Body mass index	−0.074	−0.791	0.431	0.069	0.818	0.415
Duration of AED therapy	−0.020	−0.227	0.821	0.515	3.340	0.001*
EIAEDs	−0.122	−1.399	0.165	0.203	2.143	0.034*
Duration of EIAED therapy	0.047	0.539	0.591	−0.526	−5.764	<0.001*
Duration of non-EIAED therapy	−0.062	−0.702	0.484	0.093	1.113	0.268
AED number	0.081	0.920	0.360	0.086	1.036	0.303
hs-CRP	0.199	2.297	0.023*	0.075	0.933	0.353
Total cholesterol	0.137	1.521	0.133	0.007	0.092	0.927
LDL-C	0.086	0.964	0.337	0.003	0.036	0.972
Triglyceride	0.308	3.559	0.001*	0.070	0.818	0.415
Glycated hemoglobin (HbA1c)	0.028	0.322	0.748	0.079	0.982	0.328
Creatinine	−0.151	−1.672	0.097	0.230	2.833	0.005*

*AED, antiepileptic drug, EIAEDs, enzyme-inducing antiepileptic drugs, hs-CRP, high-sensitivity C-reactive protein; LDL-C, low-density lipoprotein cholesterol; BMI, body mass index*.

*The duration of AED therapy was considered 0 in the control group*.

*p < 0.0.5.

## Discussion

In this study, we used two non-invasive parameters, OCT and urine albumin/creatinine ratio, to examine atherosclerotic microangiopathy in patients with epilepsy. Our findings confirm that patients with chronic epilepsy who are under prolonged AED therapy, particularly with EIAEDs frequently exhibit microangiopathy, including higher risk of retinopathy and nephropathy as revealed by decreased retinal vein diameters and increased urine albumin/creatinine ratio when compared with healthy controls. Our results further show that serum creatinine levels, EIAED therapy, duration of AED therapy, and duration of EIAED therapy might contribute to the narrowing of retinal veins. In addition, a high lipid profile and elevated hs-CRP levels were associated with a high urine albumin/creatinine ratio in patients with epilepsy.

Microangiopathy is an angiopathy that affects small blood vessels; the damage is common in the small arteries, arterioles, venules, and capillaries of end organs. The reduced blood supply leads to atherosclerosis-related retinopathy and nephropathy in the retina and kidney (10) or cerebral and coronary small vessel diseases in the brain and heart (19). In addition to diabetes mellitus, which is the most common cause of microangiopathy (9), conditions, including low HDL-C plus elevated triglyceride levels, hyperhomocysteinemia, hypertension, lupus, and hemolytic uremic syndrome have been found to be correlated with microangiopathy (8, 14, 20). We (3, 5) and others (2, 4, 21–23) have identified a number of biomarkers in circulation that are related to increased risk of large-vessel atherosclerosis. These biomarkers include lipid profiles, lipoproteins, hs-CRP, homocysteine, and uric acid levels in patients with epilepsy who received prolonged AED therapy. Based on the measurement of CCA IMT, we showed that the duration of AED therapy is an important independent factor affecting CCA IMT (3, 5). Since long-term exposure to AEDs alters various vascular risk factors and contributes to atherosclerosis-related macroangiopathy, we reason that long-term exposure to AEDs may also contribute to microangiopathy.

Several studies show that microalbuminuria leads to endothelial dysfunction in renal vessels and increases the risk for atherosclerosis-related renal microangiopathy (7, 16, 24, 25). In type 1 diabetes, which is associated with high cardiovascular complications, microalbuminuria is found to increase the risk of cardiovascular disease (26). In this study, we found a significantly increased urine albumin/creatinine ratio, indicating the presence of nephropathy in patients with epilepsy who received long-term AED therapy. In addition, our analysis revealed that disturbance of lipid profiles and elevated hs-CRP levels were associated with nephropathy in patients with epilepsy.

Microangiography in the retina was previously assessed by visualizing dilated pupils on retinal photographs and classified as mild nonproliferative, moderate nonproliferative, severe nonproliferative, or proliferative retinopathy (11, 12). Retinopathy can now be assessed using OCT, a new tool that can directly measure the diameter of the retinal artery and vein for further analysis of retinal microangiography (27, 28). Of the few studies (27–29) that employed OCT to assess atherosclerotic retinopathy, the results are inconsistent. The change in retinal venular diameters might be an independent factor in predicting cardiovascular disorders (29). Using OCT, we showed that the diameter of the retinal vein was narrower than that of the retinal artery in patients with epilepsy. This result is in agreement with previous studies (27, 28) which showed that narrowing of the retinal venous diameter, while the arterial diameter remained unchanged provides early evidence of incidence and progression of atherosclerosis-related microangiopathy. It follows that our results suggest that early risk of retinal angiopathy may be presented as decreased retinal vein diameter before changes in the diameter of the retinal artery are observed in patients with epilepsy. Our OCT findings also revealed that atherosclerosis-related retinopathy, indicated by narrowing of the retinal vein diameter, may occur in patients with epilepsy under long-term AED therapy. Furthermore, in addition to contributing to retinal vein narrowing, duration of AED therapy, administration of EIAEDs, and duration of EIAED therapy were found to be significantly associated with atherosclerosis-related retinopathy in our study group. Thus, OCT might be a useful tool for early detection of retinal microangiopathy in patients with epilepsy.

Our results showed that cholesterol, LDL-C, triglyceride, and hs-CRP levels were major risk factors for microalbuminuria. Previous studies have shown that triglyceride (20, 30) and hs-CRP (7, 16) levels are correlated with the structure and function of blood vessels and increased microalbuminuria. Atherosclerosis is a process that involves intravascular development of a chronic inflammatory condition that results from local interactions between modified lipoproteins, inflammatory cells, and cytokines within the walls of veins and arteries (7, 31). Our results, therefore, suggest that chronic inflammatory reactions and lipid disturbances in circulation may contribute to microangiopathy in patients with epilepsy.

Hypertension, hyperlipidemia, and diabetes are traditional risk factors attributable to disturbance of retinal microcirculation and change in retinal vessel diameters (10, 27, 32, 33). Recent evidence showed that the signs of retinopathy, including changes in retinal microvasculature, may be a risk marker for stroke and coronary microvascular disease (34, 35). However, whether retinal microvasculature may predict risk of atherosclerosis in patients with epilepsy under prolonged AED therapy remains unclear. In this study, age, serum creatinine, EIAEDs, duration of AED therapy, and duration of EIAED therapy were found to be correlated with narrow retinal vein diameter. We are aware that age may also influence the diameter of retinal vessels (27). However, after adjustment for age, the duration of AED therapy, administration of EIAEDs, and duration of EIAED therapy remain significantly correlated with the change of retinal vein thickness and vascular factors. On the other hand, non-EIAED therapy did not affect the retinal vein thickness and vascular risk profiles. We noted that the duration of therapy was 14.5 ± 12.4 years in patients who received EIAEDs with or without combination of other AEDs, the duration of non-EIAED therapy in patients without combination of EIAEDs was 13.7 ± 6.4 years. Since the duration of therapy between the EIAED and non-EIAED groups was similar, we reason that EIAEDs may play an important role in the pathogenesis of change in retinal microvasculature. Thus, our results suggest that the duration of AED treatment and the enzyme inducers may contribute to the atherosclerosis-related retinopathy in patients with epilepsy. We further propose that retinal microvascular abnormalities and microalbuminuria may be biomarkers for the screening of the risk of atherosclerosis-related diseases in patients receiving prolonged EIAED therapy.

In our previous studies (3, 5), we found that patients under long-term AED therapy may show disturbances in lipid profiles, total homocysteine levels, and folate metabolism, and increased inflammatory reaction and oxidative stress. Whereas, microangiopathy may lead to both atherosclerosis-related retinopathy and nephropathy, these events may not occur at the same time. In general, retinopathy and microalbuminuria are well-known microvascular complications, but they have different pathophysiologies and risk factors. Taking together with the risk factors with microalbuminuria, it is reasonable to suggest that atherosclerosis-related microangiopathy in patients with epilepsy is effected by long-term AED therapy, particularly with EIAEDs, in association with disturbances in cholesterol, triglyceride, homocysteine or folate metabolism, and elevation of the inflammation marker hs-CRP. Thus, CYP450 EIAEDs should be used cautiously in patients with epilepsy who also have atherosclerosis-related vascular disorders.

The pathogenesis of microangiopathy and macroangiopathy are different (36). Microangiopathy begins earlier than macroangiopathy, has no symptoms, and progresses more rapidly than macroangiopathy (36). It thus provides novel insights into early and easy diagnosis of atherosclerosis in patients with epilepsy under long-term AED therapy, particularly with EIAEDs. Furthermore, strategies to decelerate the course of endothelium-damaging atherosclerosis due to the cumulative effects of prolonged AED therapy should be promptly initiated in patients with high-vascular risks.

In conclusion, this study shows that two microangiopathies, retinopathy and nephropathy, which are associated with high levels of triglyceride and hs-CRP, duration of AED therapy, EIAEDs and duration of EIAED in patients with epilepsy who received long-term AED therapy. This information is important to our understanding of the pathogenesis of microangiopathy in patients with epilepsy, and offers a guide for the choice of drug for patients with epilepsy under long-term AED therapy. A regular work-up of lipid profiles, metabolic status, microalbuminuria, and retinal examination should be considered, particularly in aged and high-vascular risk individuals.

## Ethics Statement

Ethical approval for this study (Ethical Committee NO100-2011B) was provided by the Chang Gung Medical Foundation Institutional Review Board.

## Author Contributions

YCC and NCC participated in the design of the study, drafted the manuscript, and performed the statistical analysis. YCC, NCC, CHC, MHT, SDC, WCT, CCC and TKL participated in the clinical evaluation of patients, and drafting of the manuscript. YCC helped draft the work and revised it critically for important intellectual content. All the authors have read and approved the final manuscript.

## Conflict of Interest Statement

The authors declare that the research was conducted in the absence of any commercial or financial relationships that could be construed as a potential conflict of interest. The reviewer CR and handling editor declared their shared affiliation.
